# Native Australian seedlings exhibit novel strategies to acclimate to repeated heatwave events

**DOI:** 10.1007/s00442-025-05704-5

**Published:** 2025-05-15

**Authors:** Philippa R. Alvarez, Rosalie J. Harris, Alicia M. Cook, Verónica F. Briceño, Adrienne B. Nicotra, Andrea Leigh

**Affiliations:** 1 School of Life Sciences, University of Technology Sydney, Broadway, PO Box 123, Sydney, NSW 2007 Australia; 2 National Seed Bank, Australian National Botanic Gardens, Clunies Ross St, Acton, ACT 2601 Australia; 3 Research School of Biology, The Australian National University, 134 Linnaeus Way, Acton, ACT 2601 Australia; 4 Marine Biology Research Division, Scripps Institution of Oceanography, University of California San Diego, 9500 Gilman Drive, La Jolla, CA USA; 5 Australian National Botanic Gardens, Clunies Ross Street, Canberra, ACT Australia

**Keywords:** Thermal tolerance, Acclimation, Heat waves, Chlorophyll fluorescence, Critical temperature, Thermal thresholds, Heat stress memory

## Abstract

Heatwaves are becoming more intense and frequent. Plant photosystem thermal thresholds can vary with species, but also shift in response to environmental triggers. Both upper and lower thresholds can acclimate to repeated heatwaves through ecological stress memory, where prior exposure primes them for subsequent events. The extent to which acclimation to repeated heat stress events varies among environmental origin and/or species is unknown. Different acclimation strategies might reflect biome of origin, or may be species-specific. For 12 species from two contrasting biomes—extreme desert and benign coastal temperate—we investigated responses to two simulated heatwaves, via shifts in upper and lower critical temperatures of photosystem II, and the difference between these thresholds, thermal tolerance breadth (TTB). Biome of origin had no effect on thermal tolerance. Observed differences among species following heat events suggested two possible acclimatory strategies. In some cases, species increased thermal thresholds during the first heatwave, but at the cost of reduced thermal tolerance during the second heatwave, a sprinter strategy. Other species acclimated to the first heatwave and further increased thermal tolerance to a second heatwave, indicative of ecological stress memory, a marathoner strategy. Synthesis: these among-species responses to heatwaves could suggest distinct vulnerabilities and resilience to repeat heat stress events, with some species having limited capacity to tolerate consecutive heatwaves, possibly as the cost of acclimation is too great, with other species having the advantage of increased tolerance via stress memory, helping them survive future stress, at least in the short-term.

**Supplementary Information:**

The online version contains supplementary material available at 10.1007/s00442-025-05704-5.

## Introduction

Climate modelling suggests that global average temperatures will increase by 2–4 °C before 2100 (Arias et al. 2021). In nature, however, the increasing number of extreme events play an important role in ecological change (Seneviratne et al. 2021; Beigaite et al. 2022), such as the rise in intense and frequent heatwaves (Cowan et al. 2014). Heatwaves have been equated with the detrimental loss of function in plants, from broad-scale ecosystem effects (French et al. 2017; Kullberg et al. 2024) to individual (Smillie and Nott 1979; Kumarathunge et al. 2019) and cellular level damage (Berry and Bjorkman 1980; O'Sullivan et al. 2013). These physiological changes to plant functionality are exacerbated when plants experience repeated heat stress events, a scenario that is becoming more common (Seneviratne et al. 2021), and is resulting in dramatic shifts in global ecology (Ruthrof et al. 2018).

One way that plants cope under repeated heatwaves is via ecological stress memory, the capacity for a past stress event to influence the physiological response to a future stress event (e.g., Ahrens et al. 2021). While the genetic and epigenetic mechanisms of stress memory have been explored in model and agricultural species (Avramova 2015; Khan et al. 2022; Zhu et al. 2023), whether stress memory is detectable as increased thermal tolerance to subsequent heatwaves in wild plant species is less clear. In addition, the majority of agricultural studies focus primarily on priming, that is, the exposure to mild heat stress that can induce heat shock proteins a few hours before another, higher temperature stress that the plant would otherwise not survive without prior priming (Hilker et al. 2016; Charng et al. 2023). The acquired thermotolerance that plants gain from this priming stimulus likely reflects their capacity to acquire stress memory (Mittler et al. 2012). However, there is a distinction between a priming event, which is a short and mild stress (Wang et al. 2017), and an event triggering ecological stress memory, which is of equal duration and intensity to the subsequent high-temperature stress (Niinemets 2010; Walter et al. 2013). Research focus on non-agricultural plant responses to repeat heatwaves has increased over recent years (French et al. 2019; Milner et al. 2023). However, almost nothing is known about the extent to which ecological stress memory is related to environmental origin (but see Ahrens et al. 2021).

Plants adapted to consistently hot climates do not always have consistently high-temperature tolerance (Curtis et al. 2016), nor does it necessarily mean that they are able to withstand extreme heatwaves (Milner et al. 2023). While tolerance adaptation to extreme biomes is well established in the animal ecology literature (Schmidt-Nielsen 1965), we have little data to assess whether plant species originating from extreme desert climates are able to cope with repeated heatwave events better than those from benign environments. Plant species from both extreme and benign biomes may have adapted to withstand heat stress over time (Curtis et al. 2014; Kunert et al. 2021; Seemann et al. 1984); however, there is also considerable evidence that effects of growth temperature or leaf temperature on thermal tolerance are large (Perez and Feeley 2020; Cook et al. 2021) and may even override the effects of climate of origin (Lin et al. 2013; Aspinwall et al. 2019; Ahrens et al. 2021). It is also important to consider that among species variation in tolerance may be more pronounced than differences among contrasting biomes (Harris et al. 2024).

Photosynthetic thermal acclimation in plants has been widely discussed in the literature since Berry and Bjorkman (1980), who describe it in terms of improved photosynthetic function induced by environmental changes. Acclimation has been associated with improved thermal tolerance in response to exposure to a variety of thermal stressors (e.g. heatwaves and cold snaps; Knight and Acklerly 2002; Way and Yamori 2014; Andrew et al. 2022), but evidence is inconsistent (Zhu et al. 2018; Kullberg and Feeley 2024). To understand the vulnerability of plants adapted to contrasting environments under multiple heatwave events, there is a need to tease out the acclimation capacity and thermotolerance acquisition via stress memory of plant species from contrasting biomes ex situ. Further, while heat tolerance is a significant concern in extreme environments, these biomes also necessitate tolerance to the other extremes, such as cold or frost tolerance. Cold tolerance has been studied with respect to distribution shifts of plants in warmer climates (Wen et al. 2018) and has been suggested to evolve faster than both heat tolerance and climate niche (Wen et al. 2023). The cost of being thermally tolerant to either hot and cold extremes may have detrimental downstream effects on a plant’s health including its’ fitness (Boinot et al. 2022; Milner et al. 2023; Lee et al. 2024) and acquired thermal tolerance (Wahid et al. 2007). Yet, almost nothing is known about how both heat and cold tolerance *concurrently* acclimate under repeated heat stress events. Such insight is important for understanding plant community responses to increasingly frequent heatwaves under climate change.

Photosystems in chloroplasts are highly susceptible to temperature changes, leaving them particularly vulnerable when exposed to extreme temperatures (Berry and Bjorkman 1980; Farquhar et al. 1980; Wahid et al. 2007). The temperature sensitivity of photosystem II (PSII) provides a way of interpreting the direct impact of heat stress on plant physiology (Maxwell and Johnson 2000). PSII function can be measured by assessing the levels of minimal chlorophyll fluorescence (*F*_0_) as the leaf is subjected to a temperature stress, creating a T-*F*_0_ curve (Smillie and Nott 1979; Bilger et al. 1984). The inflection point on a T-*F*_0_ curve—known as critical temperature (*T*_crit_)—marks the shift in fluorescence from a steady state to a rapid increase in fluorescence as temperature increases (*T*_crit-hot_) or decreases (*T*_crit-cold_). *T*_crit_ is a useful indicator of potential impairment of function with downstream effects. *T*_crit-hot_ and *T*_crit-cold_ generally are used independently to assess heat tolerance *or* cold tolerance (Knight and Ackerly 2002; Arnold et al. 2021; references in Geange et al. 2021; Coast et al. 2022). The concurrent measurement of heat and cold tolerance and the difference between the two, the thermal tolerance breadth (TTB), has recently been used to characterise plant vulnerability to both temperature extremes (Sunday et al. 2019; Harris et al. 2024). What is lacking in the literature is insight into how plants shift TTB after a heatwave, i.e., whether there is evidence of ecological stress memory when exposed to a second heatwave after a period of recovery.

This study addressed the impact of repeated simulated heatwaves and intervening recovery periods on thermal tolerance and acclimation of 12 species representing two contrasting biomes—desert and coastal temperate—grown under common conditions. We measured *T*_crit-hot_, *T*_crit-cold_ and TTB, following a post-heatwave recovery period, a second heatwave and a final recovery period. Our first aim was to understand whether ecological stress memory influenced narrowing or widening of TTB between biomes and among species in response to subsequent heatwaves and periods of recovery. The presence of ecological stress memory would be suggested if TTB deacclimated (became narrower) after the first heatwave, but after the second heatwave became relatively broader, with *T*_crit-hot_ getting hotter and *T*_crit-cold_ getting colder. Our second aim was to understand whether thermal thresholds and the trajectory of how these changed between heatwave and recovery periods differed between biome and/or among species. We expected to see greater evidence for ecological stress memory in some species than others, indicating different capacities to withstand repeated heatwaves and maintain, or improve, their tolerance to heat stress in the future.

## Materials and methods

### Study species

Twelve species were selected as representatives from two contrasting biomes, six native to Australian desert systems and six originating from benign coastal temperate rainforest habitats (Table 1). Seedlings were grown from seed sourced from each biome with pots watered daily and kept in 25/15 °C (day/night) glasshouses in natural light conditions at the Australian National University (ANU). Well-established seedlings (4 cm^2^ pots) were 3–5 months old at the time of the experiment and sexually immature (Harris et al. 2024).

**Table 1:** The 12 species used in the experiment

Biome	Family	Species
Extreme—desert	Capparaceae	*Capparis mitchellii*
	Casuarinaceae	*Casuarina pauper*
	Fabaceae	*Acacia salicina*
	Fabaceae	*Acacia victoriae*
	Myrtaceae	*Eucalyptus largiflorens*
	Rutaceae	*Flindersia maculosa*
Benign—coastal temperate	Asparagaceae	*Lomandra longifolia*
	Cyperaceae	*Carex appressa*
	Fabaceae	*Acacia longifolia*
	Myrtaceae	*Melaleuca hypericifolia*
	Pittosporaceae	*Pittosporum undulatum*
	Proteaceae	*Banksia integrifolia*

### Growth conditions

During the four-week period, seedlings of each species were randomly divided between two Conviron plant growth chambers (Model PCG20) at the Plant Phenomics Facility at the Commonwealth Scientific and Industrial Research Organisation (CSIRO), Canberra. Both chambers were set to 15 °C during the night and ramped up gradually to reach the peak temperature between 1 and 4pm. In the control chamber (no simulated heatwaves), the temperature reached 25 °C and in the simulated heatwave chamber 40 °C. Leaves were selected randomly from each plant with care taken to choose leaves of intermediary age (not old or young). Light intensity was also ramped in the chamber from 0 to 800 µmol m^−2^ s^−1^ PAR during the middle of the day. Seedlings were watered before and after simulated heatwave exposure to minimise dehydration. During the intervening recovery period, seedlings were returned to the ANU glasshouses.

### Simulated heatwave treatment

**Fig. 1:**
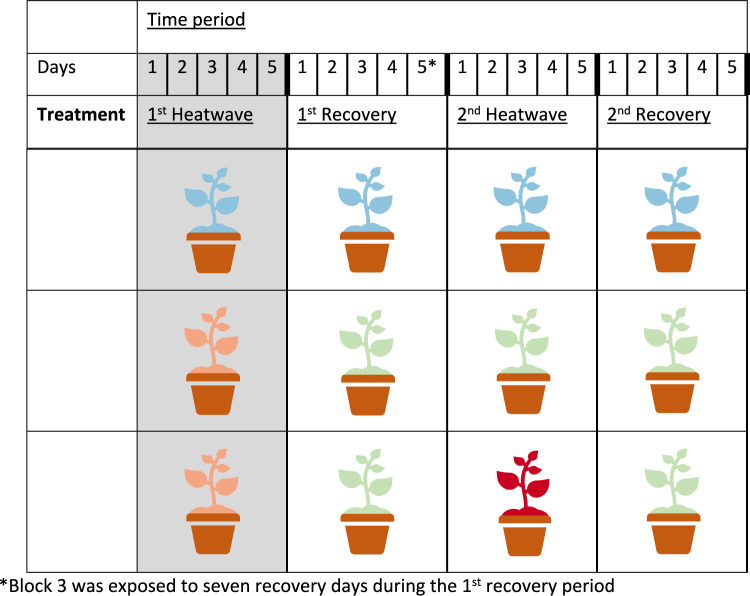
Visual summary of the experiment, indicating time period (underlined) and treatment (bold) shown. The seedlings exposed to treatment are shown in four colours: no heatwave (blue), one heatwave (peach), two heatwaves (dark red) and recovery in the glasshouse (green). Each treatment/recovery period was five days long, alternating between heatwave and recovery period. At the end of each time period (bold line after day 5), *Tcrit* measurements were taken across all three treatments. Information collected by Harris et al*.* (in review) at 1 st heatwave time period is shaded in grey

There were three treatment groups: (1) no heatwave, (2) one heatwave and (3) two heatwaves. Each thermal tolerance assay was taken after four separate time periods: 1 st heatwave, 1 st recovery, 2nd heatwave, 2nd recovery. These time periods correspond with whether the seedlings were in a growth chamber experiencing no heatwave or a heatwave, or whether they were in the glasshouse recovering (Fig. 1). Each heatwave ran for a 5-day period, both followed by a 6-day period of recovery. A control (no heatwave) was used across time periods to account for temporal changes in individual seedlings.

For clarity, the plants that were measured after both one heatwave and two heatwaves were the same for the first two time periods, both having only been exposed to one heatwave by that time point. During the 1 st recovery period, this large group of seedlings split into a one heatwave group and two heatwaves group. Due to space constraints in the chambers, the seedlings were split into five replicate blocks and staggered temporally with 1–3 days difference between the start of each block. Due to technical issues, block 1 and 2 experienced a 2-day period of underwatering during the second heatwave, block 3 had a longer first recovery period than the other blocks, and block 4 and 5 were underwatered during their first recovery period. No visible damage was observed, but to account for this potential source of variation, block was included as a random effect in the models, see below.

### Thermal tolerance assays

Photosystem II thermal tolerance was determined with chlorophyll fluorescence heat and cold assays. Leaf material collected from seedlings two hours prior to the heatwave, which was between 10:00 am and noon, or at the same time of day during the recovery period post heatwave. Leaf tissues were cut into 1 cm by 1 cm squares, randomised and placed onto a 8 × 6 grid on a thermoelectrically controlled Peltier plates (CP-121HT; TE-Technology, Inc., Michigan, USA; 152 × 152 mm surface). Underneath each leaf tissue sample, a type-T thermocouple (40-gauge, Omega Engineering) measured the tissue temperature every 5 s recorded by 48 channel dataTaker DT85 (Lontek, Australia) to measure leaf tissue temperature and account for any variation in the temperature of the Peltier plate. A layer of double-glazed glass was placed on top of the samples to ensure they were flat and reduce dehydration. Chlorophyll fluorescence was measured with a Pulse Amplitude Modulated (PAM) imaging system2 (Maxi-Imaging-PAM; Heinz Walz GmbH, Effeltrich, Germany) mounted above the Peltier plate. Leaves were dark-adapted for 15 min to obtain basal fluorescence (*F*_*0*_) with a continuous weak pulse modulating blue light (0.5 μmol photons m^−2^s^−1^). Then, a saturating pulse of 4000 μmol photons m^−2^ s^−1^ was applied for 720 ms to measure maximal fluorescence (*F*_*m*_), after which seedlings were dark adapted for a further 15 min. Variable fluorescence (*F*_*v*_) was calculated as *F*_*m*_—*F*_*0*_ to derive *F*_*v*_/*F*_*m*_ (maximum quantum yield of photosystem II) used to assess the starting function of individual leaf tissue. A weak blue pulse-modulated light measured *F*_*0*_ at 20 s intervals during the Peltier plate temperature ramp to generate a *T- F*_*0*_ curve. Temperature was obtained from the thermocouples under each leaf tissue. For heat tolerance assays, the Peltier plate was ramped from 20 °C to 65 °C at 30 °C/hr to measure *T*_crit-hot_. For cold tolerance assays, the Peltier plate ramped from 15 °C to − 20 °C at 15 °C/hr to measure *T*_crit-cold_. *T*_crit_ was calculated using the inflection point between the fast and slow rise phases of the *T- F*_*0*_ curve (Knight and Ackerly 2002) using a script adapted from Arnold et al. (2021) (https://github.com/pieterarnold/Tcrit-extraction). Thermal tolerance breadth was calculated as the difference between *T*_*crit-hot*_ and *T*_*crit-cold*_ in °C measured per plant replicate (*n* = 5).

### Statistical analysis

All analyses were conducted using R version 4.2.3 (R Core Team 2023). Effects of heatwaves and recovery periods on *T*_crit-hot_, *T*_crit-cold_, and TTB were assessed using linear mixed-effects models. Two main effects models were run to accommodate between-biome differences and among-species variations. These models were run separately as the complexity of the random effects and reduced replication were not conducive to a single, main effects model. The linear model fixed effects included either biome (coastal temperate and desert) or species (12 levels, six from each biome) as well as treatment (no heatwave, one heatwave and two heatwaves) and time period (Fig. 1; 1 st recovery, 2nd heatwave and 2nd recovery). For the random effects, block (five replicates), plant ID number and the aforementioned two-day period of heat and water stress were included. These models were performed using the LMER package in R; Bates et al. 2014). In the biome model, species was included as a random effect. The first heatwave time period was excluded from the analysis as this was discussed in Harris et al. (2024).

We next aimed to gain deeper insights into how species differed in their interaction plots in response to prior treatments. We analysed the difference in thermal tolerance breadth (ΔTTB), Δ*T*_crit-hot_ and Δ*T*_crit-cold_ among seedlings exposed to a heatwave treatment compared to those not exposed to a heatwave treatment, for each time period, as described by the following:

$$\Delta {\text{TTB}}\, = \,{\text{treatment group }}\left( {\text{1 HW or 2 HW}} \right){-\!\!-}{\text{control group }}(0{\text{ HW}})$$

A positive ΔTTB value indicated that TTB was wider in the treatment group than the control and a negative value indicated that TTB was narrower in the treatment group than the control.

Δ*T*_crit-hot_ = treatment group—control group

Δ*T*_crit-cold_ = treatment group—control group

A positive Δ*T*_crit-hot_ or Δ*T*_crit-cold_ value indicated that *T*_crit_ was hotter for Δ*T*_crit-hot_ (or cooler for Δ*T*_crit-cold_) in the treatment group than the control and a negative value indicated that *T*_crit_ was cooler in the treatment group than the control. We used these values to determine the slope of the (ΔTTB), Δ*T*_crit-hot_ and Δ*T*_crit-cold_ to ascertain if species differed in the trajectory of change in these parameters using analysis of variance (ANOVA). Models were compared using the Akaike information criterion (AIC); those with the lowest AIC value and that best fit the assumptions were chosen. All model assumptions for normality and homogeneity of variances were assessed graphically using residual plots, histograms, scatterplots and boxplots. If required, Tukey HSD post hoc tests were run using the EMMEANS R package (Lenth 2020). Figures made with GGPLOT2 (Wickham 2016).

## Results

**Fig. 2:**
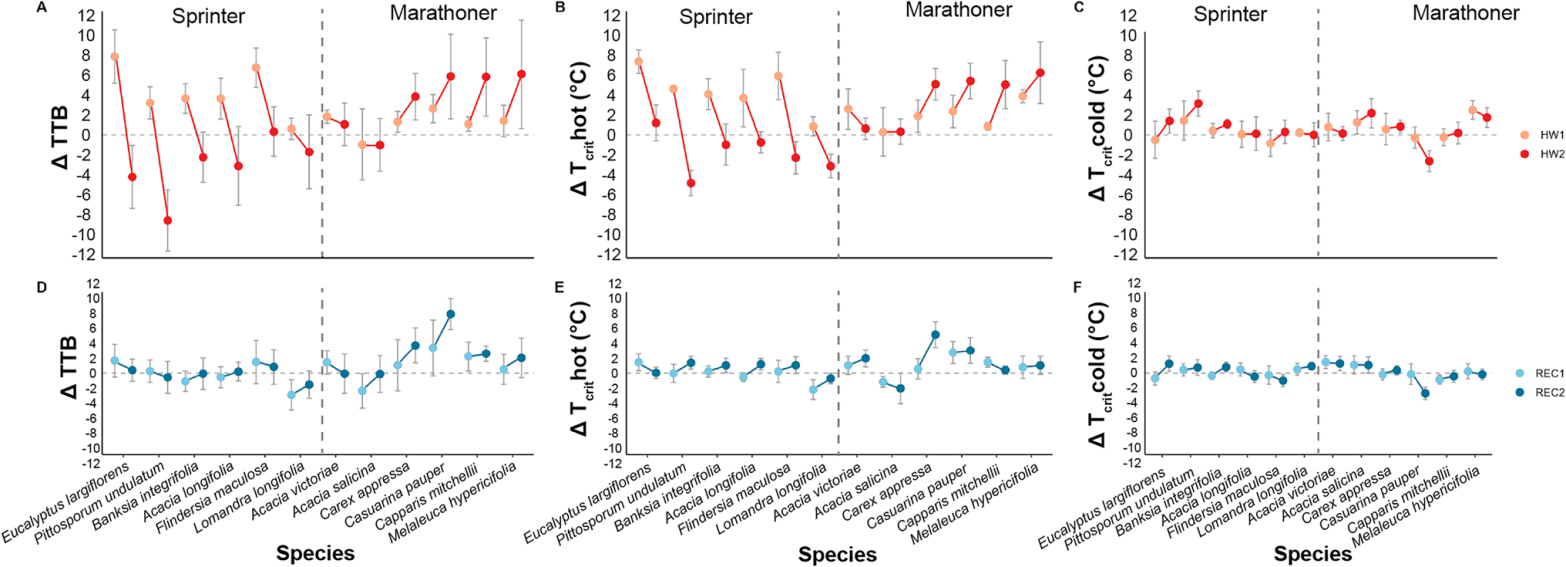
Difference between the control and treatment in species TTB (ΔTTB, °C, panel **A**), *T*crit-hot (Δ*T*crit-hot, °C, panel B) and *T*crit-cold, (Δ*T*crit-cold, °C, panel C), respectively) at each time period: first heatwave (HW1, orange symbols) and second heatwave (HW2, red symbols) (panels **A**, **B**, **C**); first recovery (REC1, light blue) and second recovery (REC2, dark blue) (panels **D**, **E**, **F**). Time periods are connected by a solid line to indicate direction. Species in panels **B**–**F** are ordered by patterns represented in panel **A**. Black vertical dashed line denotes the split between the sprinter and marathoner groups among species. S.E bars in grey

To understand if there was any influence of biome of origin on thermal tolerance thresholds, we first compared biomes with species as a random factor and found no significant differences for any of the three metrics (TTB, *T*_crit-hot_ or *T*_crit-cold_) between seedlings native to an extreme and benign biome (*F* = 0.796_1,10_, *p* = 0.783); Table 2 A). There was a significant difference in thresholds among time periods for TTB (*F* = 3.383_2,210_, *p* = 0.036) and among treatments for *T*_crit-hot_ (*F* = 6.212_2,504_, *p* = 0.002) but these were not driven by biome (Table 2 A). Looking at the differences among species after one recovery period, a second heatwave, and a second recovery period, we found that thermal tolerance breadth (TTB) varied significantly (Table 2 B). There was also a significant difference among time periods (first recovery period, a second heatwave, and a second recovery period) or as a function of heatwave treatment. *T*_crit-hot_ and *T*_crit-cold_ both varied significantly among heatwave treatments (one, two or no heatwave), and in the same direction, with thresholds generally increasing to warmer temperatures, leading to relatively stable TTB (Table 2 B). We also found significant interactions for TTB between species and time period as well as species and treatment, driven by *T*_crit-hot_. To explore the species-level responses, we plotted the difference between thermal tolerance breadth (ΔTTB), Δ*T*_crit-hot_ and Δ*T*_crit-cold_ among seedlings exposed to a heatwave treatment compared to those not exposed to a heatwave treatment. Within each time period, the significant species by treatment interaction we found for TTB and *T*_crit-hot_ (Table 2 B) shows two distinct strategies we have termed sprinters and marathoners (Fig. 2A). These distinct groups were significantly different from one another based on the slope between the treatment and the control for each species (*F* value = 19.95_1,49_, *p *value = 0.000). Species that demonstrated a sprinter response had TTB widened relative to the control after one heatwave and then narrowed so it was relatively closer to control after the second heatwave (Fig. 2A left side on x axis; *E. largiflorens*, *P. undulatum, B. integrifolia, A. longifolia*, *F. maculosa* and *L. longifolia*). The TTB for the marathoner response was initially quite similar to the control after one heatwave and then widened TTB after the second heatwave (*A. victoriae*, *A. salicina, C. appressa*, *C. pauper, C. mitchellii* and *M. hypericifolia*). This pattern also was seen in Δ*T*_crit-hot_, but not in Δ*T*_crit-cold_, which had relatively similar *T*_crit_ values between the treatment and control groups (Fig. 2B, C).

**Table 2:** Linear mixed-effects model of the relative influences on thermal tolerance breadth (TTB), *T*_crit-hot_ and *T*_crit-cold,_ of A) biome (benign and extreme) and B) species (12), on time period (first recovery period, second heatwave and second recovery period) and treatment (no heatwave, one heatwave and two heatwaves)

A	TTB			*T*_crit-hot_			*T*_crit-cold_		
	*F* value	*Df*	*p *value	*F* value	*df*	*p *value	*F *value	*df*	*p *value
Biome	0.796	1, 10	0.783	0.026	110	0.874	0.143	1, 10	0.713
Time period	3.383	2, 210	0.036**	2.860	275	0.063	5.877	2, 505	0.003
Treatment	0.754	2, 507	0.471	6.212	2504	0.002**	3.235	2, 505	0.040
Biome × Time period	1.548	2, 507	0.213	1.226	2504	0.294	0.713	2, 505	0.491
Biome × Treatment	1.441	2, 507	0.237	1.053	2504	0.350	0.770	2, 505	0.463
Time period × Treatment	0.532	4, 507	0.712	0.650	4504	0.627	0.866	4, 505	0.484
Biome × Time period × Treatment	1.417	4, 507	0.227	0.949	4504	0.435	1.791	4, 505	0.129
	Marginal *R*^2^: 0.039			Marginal *R*^2^: 0.052			Marginal *R*^2^: 0.055		
	Conditional *R*^2^: 0.213			Conditional *R*^2^: 0.208			Conditional *R*^2^: 0.288		

B	TTB			*T*_crit-hot_			*T*_crit-cold_		
	*F* value	*df*	*p* value	*F* value	*df*	*p* value	*F* value	*df*	*p* value
Species	2.625	11, 427	0.003**	2.317	11, 424	0.009**	2.486	11,428	0.005**
Time period	3.879	2, 218	0.023*	3.522	2, 424	0.031*	6.093	267	0.003**
Treatment	0.753	2, 427	0.472	6.274	2, 424	0.002**	3.189	2428	0.043*
Species × Time period	1.776	22, 427	0.017*	1.992	22, 424	0.005**	1.305	22,428	0.164
Species × Treatment	1.938	22, 427	0.007**	2.158	22, 424	0.002**	0.942	22,428	0.539
Time period × Treatment	0.654	4, 427	0.624	0.786	4, 424	0.535	0.916	4428	0.454
Species × Time period × Treatment	1.161	44, 427	0.237	1.188	44, 424	0.200	0.899	44,428	0.656
	Marginal *R*^2^: 0.236			Marginal *R*^2^: 0.257			Marginal *R*^2^: 0.198		
	Conditional *R*^2^: 0.441			Conditional *R*^2^: 0.440			Conditional *R*^2^: 0.424		

All models for biome (**A**) included block and species as random effects, and for species (**B**) included block and plant ID number. Data for the first heatwave are from Harris et al*.* (2023). The level of significance is also indicated (*p < 0.05, **p < 0.01)

As for the recovery periods, most species had returned to control levels after recovering from the first and second heatwaves, and thus there were significant time period effects or interactions, with the exception of the marathoner group which displayed marginal differences between treatments and controls for ΔTTB and Δ*T*_crit-hot_ (Fig. 2D–F).

## Discussion

This study sought to understand whether native plant species exhibited ecological stress memory in photosystem thermal tolerance thresholds (TTB, *T*_crit-hot_ and *T*_crit-cold_) after exposure to simulated heatwaves and recovery periods. We expected to find significant differences among the time periods and treatment groups and found there were significant differences in tolerance thresholds among plants that had just experienced a recovery vs heatwave period and between plants subjected to one vs two heatwaves. TTB was significantly different across time periods, but not among treatments, suggesting that while *T*_crit-hot_ and *T*_crit-cold_ shifted in response to heatwave treatments, they did so in a similar manner, therefore, not changing thermal tolerance breadth significantly. One aspect we did not anticipate was two distinct acclimation/deacclimation response patterns (Fig. 2). Below, we explore the lack of biome effect, species thermal tolerances and the response trajectories (‘strategies’) exhibited by plants after the repeated heatwaves.

### Lack of differences among biomes

The lack of significant difference in TTB between plants adapted to an extreme desert vs benign coastal temperate environments, even within the control treatment, seems counterintuitive, yet convergence of single heat tolerance thresholds under common conditions has been observed before. Using the same species as the current study, Harris et al. (2024) found that biome had a minimal effect on thermal tolerance metrics in response to hot days, cold nights or a combination of both. They found that, regardless of whether a plant was originally from a desert, temperate or alpine environment, TTB narrowed when juvenile plants were exposed to both hot days and cold nights (Harris et al. 2024). The negligible influence of biome may be a result of the common growth environment the juveniles were raised in. Another study focusing on the western USA desert and coastal congeneric species grown in a common environment also found negligible differences in heat tolerance between biome of origin (Knight and Ackerly 2003). Those authors interpreted the lack of differentiation among biomes to reflect the acclimatisation of the plants to their common growth conditions, overshadowing any effect of where the species originated. In the same study, significant differences in heat thresholds between biomes were found when measurements were conducted on plants in the field (Knight and Ackerly 2003), which has also been observed in more recent Australian studies (O’Sullivan et al. 2017; Briceño et al. 2023). In contrast, others have found upper threshold temperatures of the common grown species from a single desert biome to vary by 4–7 °C (Downton et al. 1984; Curtis et al. 2016). In the current study, we found only a 2 °C difference in *T*_crit-hot_, a non-significant difference that supports the idea that prevailing conditions can have a strong influence on thermal tolerance acclimation relative to biome of origin (Knight and Ackerly 2003; Slot and Kitajima 2015).

The overall negligible influence of the biome of origin on our study species thermal thresholds was also reflected in responses to successive treatments, where we found no significant differences in TTB between biomes after exposure to heatwave and recovery periods. Interestingly, Ahrens et al., (2021) found that *Corymbia calophylla* genotypes from a warmer climate of origin had significantly different responses to a moderate heatwave compared with those of cooler climate genotypes. However, these differences between warmer and cooler climate of origin disappeared with severe and more frequent heatwaves (Ahrens et al. 2021). These findings further support our conclusion for thermal tolerance breadth, that common growth conditions can diminish the influence of the climate of origin when it comes to acclimation to repeated heat stress.

### Species distinct heatwave responses: sprinters v marathoners

We found significant differences in TTB among species in response to repeated heatwaves. Importantly, when comparing responses after both the heatwave and recovery periods, significant species by treatment interactions were found for plants exposed to one heatwave versus two heatwaves (Fig. 2). In eliciting a response to successive treatment exposures, our plants appeared to adopt one of two main strategies, which we describe as that of a sprinter or marathoner.

For the sprinter group, TTB was markedly wider than the control after the first heatwave (high, positive ΔTTB) and mostly narrower than the control (negative ΔTTB) after the second heatwave (Fig. 2A). This pattern of response may suggest that seedlings of these species were able to acclimate to the first heatwave, but could not sustain that response after a second heatwave, where we observed a relative decrease in thermal tolerance. This shift indicates that sprinters would have a reduced capacity to protect against, and therefore potentially be more vulnerable to, repeated heat stress. Further investigation might increase the challenge to these thermal thresholds under more severe conditions to determine if these species are vulnerable to future intensification of heatwaves or if they are, instead, super-pacers, which take heatwaves in their stride.

For the marathoners, TTB was closer to the control group after the first heatwave and then wider after the second heatwave. The response of these species suggests that the first heatwaves may have triggered a degree of ecological stress memory in the seedlings, preparing them for increased tolerance to subsequent heat events. The capacity for this more resilient group to acclimate through ecological stress memory is said to be a common occurrence in plants (Walter et al. 2013; Ahrens et al. 2021). However, such a response represented only half of our 12 species, with the rest suffering a dramatic drop in thermal thresholds in response to a second heatwave. Using a metric like *T*_crit_ to assess this vulnerability denotes potential impairment of function initially, which may later have downstream effects on plant acclimation as a whole. With this understanding, it is important not to understate the potential shifts in ecosystem dynamics as a result of species differences in thermal acclimation strategy under repeated heatwave scenarios.

### Differences among species during recovery

Interestingly, the change in ΔTTB between the recovery periods was small when compared to the large differences seen between heatwaves (Fig. 2D). Irrespective of which strategy plants displayed to cope with heatwaves, for the most part, their thermal thresholds deacclimated quickly by returning to baseline levels after exposure to each heatwave. When acclimation occurs in response to an environmental stimulus, a plant can achieve improved photosynthetic performance (Berry and Bjorkman 1980). However, this improved performance comes with an energetic cost as the production of ATP for PSII protection and repair during stress itself requires a number of ATP-dependent events (Murata and Nishiyama 2018). Returning thermal tolerance thresholds to baseline levels during subsequent benign conditions would conserve energy and support the capacity for acclimation to future heat stress events. Notably for this study, the ability to deacclimate occurred regardless of the acclimation strategy employed by the species to survive the second heat stress.

In partial agreement with our findings, Ahrens et al. (2021) found that multiple heatwave events altered recovery, dependent on the climate of origin, with a small but significant difference between the two recovery periods for certain species, as observed for the recovery of the marathoner group in our study. In these species,* T*_*crit-hot*_ remained higher, and TTB wider, than baseline levels after the post-heatwave stress, especially with *Casuarina pauper* and *Carex appressa* (Fig. 2D, E). One explanation as to why these species are showing signs of potential delayed deacclimation of TTB during the recovery period could again reflect stress memory and acquired acclimation to prevent future damage. Mechanistically, genetic expression of a variety of heat shock proteins and factors during periods of heat stress can enable plants to maintain photosynthetic thermal tolerance thresholds for longer periods of time (Lin et al. 2014; Wu et al. 2013; Charng et al. 2023). Species-specific differences in heat shock protein expression may explain the reduced TTB deacclimation of some marathoner species.

### Implications and future considerations

Our study has shown that some plant species are able to acclimate to an initial heat stress event and be more thermally tolerant to a second heatwave. Other species show signs of not having the capacity to acclimate for a second heatwave, potentially leaving them particularly vulnerable to what is now becoming the norm—repeated heatwave events in quick succession. These findings have implications for future ecosystem dynamics, including shifting species composition and likely invasion from hardier species that illicit a stress memory response and continue to function during repeated heatwave events. To better understand whether the two response types identified here (sprinter, marathoner) hold as adaptive strategies across a broader species set, we suggest the examination of short- and long-term acclimation relative to realistic thermal regimes. That acclimation is an energy intensive process is shown by the sprinter group through the reduced capacity to recover after a second heatwave event. As might be expected for plants undergoing heat stress, these responses are by far the strongest for *T*_crit-hot_, which shifts substantially compared to *T*_crit-cold._ Nonetheless, cold tolerance has older evolutionary origins than heat tolerance (Wen et al. 2018) and so may simply have a more stable baseline. The energy requirements of heat vs cold tolerance are something to consider in the future.

## Supplementary Information

Below is the link to the electronic supplementary material.

Supplementary file1 (DOCX 30 KB)

## Data Availability

The R scripts and datasets used to conduct the data analyses are available at the DRYAD Digital Repository (10.5061/dryad.vdncjsz62).
