# Supplementary material for: Native Australian seedlings exhibit novel strategies to acclimate to repeated heatwave events
Source: Oecologia. 2025 May 15;207(6):84. doi: 10.1007/s00442-025-05704-5 (PMC12081561; doi:10.1007/s00442-025-05704-5)
Supplement: Supplementary file 1 — Supplementary file1 (DOCX 30 KB) [file 442_2025_5704_MOESM1_ESM.docx]

Table S1. Mean *T*_crit-hot,_ *T*_crit-cold_ and TTB for each species at each time period, comparing control and treatment, ± standard error. Treatment: HW1 = 1 HW exposure, Recovery 1 = 1 HW exposure, HW2 = 2 HW exposures and Recovery 2 = 2 HW exposures.

|  |  |  |  | *T*_crit-hot_ | |  | *T*_crit-cold_ | |  | TTB | | |
| --- | --- | --- | --- | --- | --- | --- | --- | --- | --- | --- | --- | --- |
| Biome | Species | Time period |  | Control | Treatment |  | Control | Treatment |  | Control | Treatment |  |
| Benign | *Acacia longifolia* | HW1 |  | 42.3 ± 2.1 | 45.9 ± 1.3 |  | -12.4 ± 1 | -12.3 ± 1 |  | 54.7 ± 2.1 | 58.2 ± 1.1 |  |
|  |  | Recovery 1 |  | 48 ± 0.6 | 47.7 ± 1.3 |  | -12.9 ± 1.1 | -12.5 ± 1.7 |  | 60.8 ± 1.1 | 60.2 ± 2.5 |  |
|  |  | HW2 |  | 46.2 ± 0.7 | 45.2 ± 1.7 |  | -10.6 ± 2.1 | -8.5 ± 1 |  | 56.8 ± 2.4 | 53.8 ± 2.7 |  |
|  |  | Recovery 2 |  | 45.9 ± 0.7 | 46.8 ± 0.8 |  | -10.3 ± 1.4 | -9.6 ± 0.5 |  | 56.2 ± 1.8 | 56.5 ± 0.6 |  |
|  | *Banksia integrifolia* | HW1 |  | 43.2 ± 0 | 46.1 ± 1.1 |  | -10.7 ± 0.3 | -9.9 ± 0.6 |  | 53.8 ± 0 | 56 ± 1.4 |  |
|  |  | Recovery 1 |  | 46.1 ± 1.9 | 45.9 ± 0.9 |  | -10.4 ± 0.3 | -9.5 ± 0.5 |  | 56.6 ± 2.1 | 55.4 ± 1.1 |  |
|  |  | HW2 |  | 44.7 ± 2.3 | 44 ± 1.4 |  | -11.3 ± 0.3 | -9.8 ± 0.5 |  | 56 ± 2.6 | 53.8 ± 1.6 |  |
|  |  | Recovery 2 |  | 46.3 ± 1.6 | 47.7 ± 0.7 |  | -10.8 ± 1 | -9.4 ± 0.6 |  | 57.1 ± 2.5 | 57.1 ± 0.4 |  |
|  | *Carex appressa* | HW1 |  | 50.1 ± 0.8 | 51.9 ± 1.2 |  | -16.2 ± 1 | -15.6 ± 1.8 |  | 66.3 ± 1.5 | 67.6 ± 1.3 |  |
|  |  | Recovery 1 |  | 48.4 ± 0.8 | 48.8 ± 2.9 |  | -14 ± 1.3 | -14.8 ± 0.9 |  | 62.4 ± 2.1 | 63.6 ± 3.5 |  |
|  |  | HW2 |  | 43.6 ± 1.9 | 49.6 ± 1.2 |  | -12.4 ± 0.3 | -10.9 ± 1 |  | 56 ± 2.1 | 60.5 ± 1.1 |  |
|  |  | Recovery 2 |  | 42.2 ± 2.5 | 47.8 ± 0.5 |  | -12.3 ± 0.4 | -10.9 ± 1 |  | 54.5 ± 2.5 | 58.6 ± 0.8 |  |
|  | *Lomandra longifolia* | HW1 |  | 49.5 ± 1.1 | 50.3 ± 0.3 |  | -13.1 ± 0.8 | -12.9 ± 0.7 |  | 62.6 ± 0.8 | 63.2 ± 0.5 |  |
|  |  | Recovery 1 |  | 49.7 ± 1.1 | 46.5 ± 1 |  | -12.9 ± 0.5 | -12.9 ± 1.1 |  | 62.5 ± 1.3 | 59.3 ± 1.9 |  |
|  |  | HW2 |  | 48.3 ± 1.6 | 46 ± 1.8 |  | -11.9 ± 0.9 | -12.6 ± 1.2 |  | 60.2 ± 1.9 | 58.6 ± 2.9 |  |
|  |  | Recovery 2 |  | 46.5 ± 0.4 | 45.9 ± 1.3 |  | -11.5 ± 0.6 | -10.4 ± 0.6 |  | 58 ± 0.9 | 56.3 ± 1.6 |  |
|  | *Melaleuca hypericifolia* | HW1 |  | 44.4 ± 0.7 | 48.1 ± 0.7 |  | -11.2 ± 0.6 | -8.8 ± 0.9 |  | 55.6 ± 0.9 | 56.9 ± 1.2 |  |
|  |  | Recovery 1 |  | 45.2 ± 1.4 | 45.6 ± 1.4 |  | -11.4 ± 1.2 | -11.6 ± 0.7 |  | 56.6 ± 1.3 | 57.1 ± 1.4 |  |
|  |  | HW2 |  | 40.1 ± 2.2 | 47.7 ± 2.1 |  | -12.8 ± 1.1 | -11.1 ± 0.9 |  | 52.9 ± 2.6 | 58.8 ± 2.9 |  |
|  |  | Recovery 2 |  | 46.8 ± 1.5 | 48.4 ± 0.9 |  | -11.3 ± 0.6 | -11.9 ± 0.9 |  | 58.1 ± 1.4 | 60.4 ± 1.6 |  |
|  | *Pittosporum undulatum* | HW1 |  | 44.5 ± 0 | 48.9 ± 0.8 |  | -10.2 ± 0.5 | -9.5 ± 0.9 |  | 54.9 ± 0 | 58.3 ± 0.7 |  |
|  |  | Recovery 1 |  | 47.3 ± 0.2 | 48 ± 1.1 |  | -11 ± 0.7 | -10.7 ± 0.6 |  | 58.4 ± 0.7 | 58.7 ± 1.5 |  |
|  |  | HW2 |  | 51 ± 1.6 | 45.7 ± 0.8 |  | -13.1 ± 1.8 | -10.1 ± 0.7 |  | 64.1 ± 2.9 | 55.8 ± 1.4 |  |
|  |  | Recovery 2 |  | 46 ± 1.3 | 47.7 ± 0.7 |  | -12 ± 1.2 | -9.7 ± 0.6 |  | 58 ± 1.5 | 57.4 ± 1.1 |  |
| Extreme | *Acacia salicina* | HW1 |  | 47 ± 1.2 | 49.9 ± 0.4 |  | -12.4 ± 0.7 | -10.8 ± 0.9 |  | 59.4 ± 1 | 60.7 ± 0.7 |  |
|  |  | Recovery 1 |  | 48.6 ± 0.6 | 47.3 ± 0.7 |  | -13 ± 1.7 | -12.3 ± 0.1 |  | 61.6 ± 2.2 | 59.6 ± 0.8 |  |
|  |  | HW2 |  | 46.9 ± 0.8 | 48.4 ± 0.5 |  | -13.1 ± 1.5 | -10.3 ± 2 |  | 60 ± 2.2 | 58.7 ± 2.3 |  |
|  |  | Recovery 2 |  | 47.2 ± 0 | 48.7 ± 0.6 |  | -12.5 ± 1.1 | -11.6 ± 1.2 |  | 59.7 ± 0 | 60.2 ± 1.3 |  |
|  | *Acacia victoriae* | HW1 |  | 45.7 ± 0 | 49.4 ± 1 |  | -12.3 ± 0 | -10.9 ± 0.6 |  | 58.5 ± 0 | 60.4 ± 1 |  |
|  |  | Recovery 1 |  | 47.3 ± 1.5 | 48.9 ± 0.6 |  | -12.5 ± 0.5 | -12.5 ± 1.5 |  | 59.8 ± 1.4 | 61.4 ± 2 |  |
|  |  | HW2 |  | 47.7 ± 0.6 | 49.6 ± 1.2 |  | -13.3 ± 0.4 | -13.4 ± 1.3 |  | 61 ± 0.9 | 62.9 ± 1.9 |  |
|  |  | Recovery 2 |  | 46 ± 1.6 | 47.2 ± 0.8 |  | -11.5 ± 0.8 | -10.3 ± 0.6 |  | 57.6 ± 2.3 | 57.4 ± 1.1 |  |
|  | *Capparis mitchellii* | HW1 |  | 47.8 ± 0.8 | 48.6 ± 0.9 |  | -12 ± 0.5 | -12.3 ± 0.8 |  | 59.9 ± 0.8 | 60.9 ± 1.1 |  |
|  |  | Recovery 1 |  | 46.9 ± 1.1 | 48.3 ± 0.9 |  | -11.9 ± 0.8 | -13 ± 1 |  | 58.9 ± 1.8 | 61.3 ± 1.9 |  |
|  |  | HW2 |  | 44.5 ± 1.6 | 50 ± 1.2 |  | -11.5 ± 0.7 | -9.9 ± 1.6 |  | 56 ± 2.2 | 59.9 ± 2.7 |  |
|  |  | Recovery 2 |  | 46.6 ± 0.2 | 48.1 ± 0.3 |  | -11.2 ± 1.3 | -11.5 ± 0.5 |  | 57.9 ± 1.2 | 59.6 ± 0.6 |  |
|  | *Casuarina pauper* | HW1 |  | 47.5 ± 0.9 | 49.7 ± 0.8 |  | -10.7 ± 0.8 | -10.9 ± 0.4 |  | 58.1 ± 1 | 60.7 ± 0.7 |  |
|  |  | Recovery 1 |  | 43.9 ± 2.1 | 46.9 ± 2.3 |  | -12.2 ± 1.6 | -12.9 ± 1.5 |  | 56.1 ± 3.5 | 59.8 ± 1.8 |  |
|  |  | HW2 |  | 41.2 ± 2.6 | 45.1 ± 2.6 |  | -9.8 ± 1.1 | -11.6 ± 1.2 |  | 51 ± 2.9 | 56.7 ± 3.5 |  |
|  |  | Recovery 2 |  | 44.3 ± 2.3 | 49.2 ± 1.2 |  | -9.4 ± 0.8 | -13.1 ± 0.8 |  | 53.6 ± 2.9 | 62.4 ± 1.2 |  |
|  | *Eucalyptus largiflorens* | HW1 |  | 42.6 ± 1.4 | 49.7 ± 0.9 |  | -9.2 ± 0.9 | -9.7 ± 1.5 |  | 51.8 ± 1.7 | 59.4 ± 1.1 |  |
|  |  | Recovery 1 |  | 46.7 ± 1.4 | 48.3 ± 1 |  | -10.6 ± 0.7 | -10.9 ± 1.3 |  | 57.3 ± 1 | 59.2 ± 2 |  |
|  |  | HW2 |  | 45.6 ± 1.3 | 44.6 ± 1.5 |  | -12.5 ± 0.7 | -9.4 ± 0.9 |  | 58.1 ± 1.4 | 54 ± 1.7 |  |
|  |  | Recovery 2 |  | 47.9 ± 0.3 | 48.8 ± 0.6 |  | -11.3 ± 1.2 | -10.9 ± 0.5 |  | 59.2 ± 1.4 | 59.6 ± 0.5 |  |
|  | *Flindersia maculosa* | HW1 |  | 38.8 ± 1.5 | 44.6 ± 2.2 |  | -11 ± 0.7 | -11.8 ± 0.9 |  | 49.9 ± 1.6 | 56.4 ± 1.8 |  |
|  |  | Recovery 1 |  | 44.8 ± 2 | 45.3 ± 1.3 |  | -12 ± 1.2 | -13.1 ± 1.4 |  | 56.7 ± 1.4 | 58.4 ± 2.2 |  |
|  |  | HW2 |  | 49.1 ± 1.2 | 49.9 ± 1 |  | -12 ± 0.6 | -11.5 ± 1.2 |  | 61.1 ± 1 | 61.4 ± 1.7 |  |
|  |  | Recovery 2 |  | 46.4 ± 1.2 | 46.8 ± 1.4 |  | -10.5 ± 0.6 | -11.1 ± 1.2 |  | 56.9 ± 1.6 | 57.8 ± 2.2 |  |
